# Effectiveness of mobile application interventions for stroke survivors: systematic review and meta-analysis

**DOI:** 10.1186/s12911-023-02391-1

**Published:** 2024-01-02

**Authors:** Wenjing Cao, Azidah Abdul Kadir, Wenzhen Tang, Juan Wang, Jiamu Yuan, Intan Idiana Hassan

**Affiliations:** 1 Xiangnan University, Chenzhou, Hunan Province China; 2 School of Health Sciences, Health Campus, Universiti Sains Malaysia, Kubang, Kerian, 16150 Malaysia Kelantan; 3 Department of Family Medicine, School of Medical Sciences, Health Campus, Universiti Sains Malaysia, Kubang Kerian, 16150 Malaysia Kelantan; 4 Guangzhou University of Chinese Medicine, Guangzhou, Guangdong Province China

**Keywords:** Smartphone, App, Systematic review, Interventions, Mobile health, eHealth, Stroke

## Abstract

**Background:**

Although smartphone usage is ubiquitous, and a vast amount of mobile applications have been developed for chronic diseases, mobile applications amongst stroke survivors remain unclear.

**Objective:**

This systematic review and meta-analysis aimed to determine the effectiveness of mobile applications on medication adherence, functional outcomes, cardiovascular risk factors, quality of life and knowledge on stroke in stroke survivors.

**Methods:**

A review of the literature was conducted using key search terms in PubMed, EMBASE, Cochrane and Web of Science databases until 16 March 2023 to identify eligible randomized controlled trials (RCTs) or controlled clinical trial (CCTs) of mobile application interventions among stroke survivors. Two reviewers independently screened the literature in accordance with the eligibility criteria and collected data from the articles included. Outcomes included medication adherence,functional outcomes,cardiovascular risk factors, quality of life,and knowledge of stroke.

**Results:**

Twenty-three studies involving 2983 participants across nine countries were included in this review. Sixteen trials involved health care professionals in app use, and seven trials reported measures to ensure app-based intervention adherence. Mobile applications targeting stroke survivors primarily encompassed three areas: rehabilitation, education and self-care. The participants in the studies primarily included young and middle-aged stroke survivors. Meta-analysis results demonstrated that mobile application intervention significantly improved trunk control ability (mean differences [MD] 3.00, 95% CI [1.80 to 4.20]; *P* < 0.00001), Fugl–Meyer assessment of upper extremity (MD 9.81, 95% CI [8.72 to 10.90]; *P* < 0.00001), low-density lipoprotein cholesterol (MD − 0.33, 95% CI [− 0.54 to − 0.11]; *P* = 0.003) and glycosylated haemoglobin A_1c_ (HbA_1c_)<7 levels (MD 1.95, 95% CI [1.17 to 3.25]; *P* = 0.01). However, the mobile application intervention did not differ significantly in medication adherence, 10-min walk test (10 MWT), Barthel index, systolic blood pressure, diastolic blood pressure, high-density lipoprotein cholesterol, body mass index, smoking, health-related quality of life and knowledge of stroke.

**Conclusion:**

Our study suggested that mobile application interventions may have a potential benefit to stroke survivors, but clinical effectiveness should be established. More studies using rigorous designs are warranted to understand their usefulness. Future research should also involve more older adult stroke survivors.

**Supplementary Information:**

The online version contains supplementary material available at 10.1186/s12911-023-02391-1.

## Introduction

Based on the most recent Global Burden of Disease 2019 stroke burden estimates globally, stroke is the second leading cause of death and a major cause of disability [1]. In 2019, 12.2 million (95% UI 11·0–13·6) incident strokes and 101 million (93·2–111) prevalent strokes were reported. Globally, stroke was responsible for 143 million disability-adjusted life-years and 6.55 million deaths [1]. In China, the annual number of new stroke cases is approximately 3.94 million [2]. Stroke cost the 32 European countries under analysis €60 billion, with health care accounting for €27 billion (45%), representing 1.7% of health expenditure in 2017 [3]. The estimated global cost of stroke is over US$891 billion, representing 1.12% of the global GDP [4]. Stroke remains a disease of immense public health significance in the twenty-first century despite the advances in primary and secondary prevention as well as acute stroke treatment and neurorehabilitation [5]. Furthermore, stroke has caused a huge public health burden, which is set to increase in the future because of the ageing population and increased prevalence of modifiable stroke risk factors [6].

The growing burden of stroke worldwide strongly suggests that current primary stroke and cardiovascular disease prevention strategies are either not universally adopted or not sufficiently effective [4]. Literature has demonstrated that the importance of long term rehabilitation for people with stroke is increasingly evident, howerer multi-factorial barriers resultes in lacking of long term rehabilitation services [7]. Therefore, the implementation of new approaches that are effective in reaching a wider population and promoting long-term stroke management in an economically viable way is essential to mitigate the disease burden of stroke. Recent advances in mobile (smartphone) technologies and their worldwide use (about 1.4 billion users) provide unique opportunities to elicit behaviour change for disease management [8]. Delivering care outside traditional brick-and-mortar settings has been fuelled by rapid innovation and economic growth in mobile technology development, consumer adoption as well as the coronavirus disease 2019 pandemic [9, 10]. Mobile applications are easily accessible, convenient and easily adopted, and they can promote social distancing. The increasing availability, convenience and ease of use of apps promote the growth of smartphone applications that can be used for intervention amongst stroke survivors.

Over the past decade, several trials of mobile health (mhealth) interventions for stroke survivors have been published [11, 12]. Despite the strong uptake of mhealth technology targeted at stroke survivors, whether this strategy improves patient-related outcomes remains unclear. Major knowledge gaps exist about their utility and efficacy. To our knowledge, only two narrative systematic reviews have been conducted in the area of mobile applications used amongst stroke survivors [13, 14]. However, one narrative systematic review aimed to determine the effectiveness of mobile applications in the rehabilitation of stroke survivors [13]. The other narrative systematic review has explored the role of mHealth apps supporting the self-management of health and function amongst survivors [14]. On the contrary, recent systematic review and meta-analysis have studied the efficacy of telerehabilitation in poststroke patients [15] and impact of mhealth and telehealth technology on medication adherence of patients with stroke [16]. However, these reviews included any mobile technology-based intervention, such as personal digital assistants, without focusing on mobile applications. Published evidence for the beneficial effects of mobile applications amongst stroke survivors is lacking. No existing systematic review or meta-analysis examining the effect of mobile applications interventions amongst stroke survivors has been conducted. Such a review is necessary to inform the development of scalable and effective activity interventions amongst this population. Given the increased interest of the European Society of Cardiology and the American Heart Association on the use of mHealth technologies to improve patient outcomes [17, 18], a new systematic review with an explicit focus on mobile application interventions for patients with stroke is warranted.

This systematic review and meta-analysis aims to determine the effectiveness of mobile applications on medication adherence, functional outcomes, cardiovascular risk factors, quality of life and knowledge on stroke amongst stroke survivors.

## Methods

This systematic review and meta-analysis was performed in accordance with the Preferred Reporting Items for Systemic Reviews and Meta-Analyses (PRISMA) [19] and the ‘PRISMA 2020 Checklist’ was used (Appendix S1). The protocol of this study was registered in the PROSPERO international prospective register for systematic reviews (CRD42023402378).

### Search strategy

PubMed, EMBASE, Cochrane and Web of science databases were systematically searched up to 26 May 2023 to identify relevant publications. Combinations of the key words and indexing terms such as MeSH or Emtree linked to the search domains were used. An automated electronic search was performed using the MeSH terms identified in Pubmed. The following MeSH terms and keywords were included: “stroke” OR “brain infarction” OR “transient ischemic attack” OR “cerebral hemorrhage” OR “subarachnoid hemorrhage” AND “mobile applications” OR “mobile app” OR “App-based” OR “portable software app*” OR “tablet application”, AND “randomized controlled trial” or “RCT” or “quasi-experiment” or “trial” or “intervention”. or “quasi-experiment” or “randomized clinical trial” or “controlled clinical trial”. A detailed search strategy for each database was presented in the Supplementary material online, Appendix S2. Boolean operators were used to combine and cross-reference between domains. In addition, a manual search was performed by checking the reference lists of reviews of related topics and selected articles.

### Eligibility criteria

The core elements of inclusion criteria in the PICOS format were used as follows: 1) Population: stroke survivors; 2) Intervention: intervention delivered via a smartphone application; 3) Comparison: the control group that received only the usual medical interventions and the intervention group that used the mobile application in addition to the usual medical interventions; 4) Outcomes: effects of interventions in overall or at least one type of relevant health-related outcomes (e.g., medication adherence, functional outcomes, cardiovascular risk factor, quality of life and so on); and 5) Study Design: randomized controlled trials (RCTs) or controlled clinical trial (CCTs). As the inclusion of unpublished studies itself may introduce bias [20], only publications in peer-reviewed journals were included in this systematic review and meta-analysis.

Published conference abstracts,studies that published in languages other than English, case reports, studies based on a webpage or website without apps, studies did not have sufficient information about the measurement of the outcome of interest, preprint papers, qualitative studies, letters to editors, simulation studies, studies only introducing the interface or internal structure of the apps, surveys or reviews and studies describing protocols were excluded from the review.

### Study selection and data extraction

Two researchers independently screened the identified papers to minimise possible errors and bias during the selection process. The authors first screened the abstracts of the candidate papers against the inclusion and exclusion criteria. Moreover, the authors selected the final papers for inclusion after reading the full manuscripts of the eligible papers and their references. Any disagreements were resolved through discussion amongst the authors to reach consensus.

A standardised data extraction form was used to extract the following information: first author, publication year, participant characteristics (age group,sample size and country), app (app names/devices used with the app and functionality/main features), study design, intervention and follow-up duration, involvement of health care professional (HCP), measures to ensure compliance of the participants and outcomes. The corresponding authors were contacted for unclear or missing information.

### Study quality evaluation

Two researchers independently conducted quality evaluation. Discrepancies were resolved through discussion by the two researchers, and decisions were independently assessed by a third investigator. Quality evaluation of RCTs was conducted independently by two researchers using the Cochrane collaboration’s tool for assessing risk of bias [21], which covers six domains of bias: selection bias, performance bias, detection bias, attrition bias, reporting bias and other bias. The risk of bias was determined as high, low or unclear with their corresponding causes. The quality evaluation of controlled clinical trials was conducted using the Risk of Bias Tool in Non-Randomized Studies of Interventions (ROBINS-I) [22], which covers bias caused by confounding factors, intervention classification, participant selection, deviations from intended intervention, missing data, outcome measurement and selection of reported results. The categories for risk of bias judgements include ‘low risk’, ‘moderate risk’, “serious risk” and “critical risk” of bias. The risk of bias graphs was generated using RevMan 5.4.

### Statistical analysis

All meta-analyses were performed using RevMan version 5.4 (The Cochrane Collaboration, Copenhagen, Denmark). Standardised mean difference (SMD), odds ratio (OR) and 95% confidence interval (CI) were regarded as statistical indicators. Inverse-variance-weighted linear meta-analysis of SMD (Hedge’s g) was performed to measure the effect size of mobile application on the change of review outcomes such as medication adherence/BP/LDL-C. In brief, Hedge’s g value of < 0.2 indicates a mild effect, and ~ 0.5 and > 0.8 indicate moderate and strong effect, respectively. The heterogeneity of results was assessed using the *I*^*2*^ statistical test. The random-effect model or fixed-effect model was determined on the basis of the results of the heterogeneity* I*^*2*^ test, with *I*^*2*^ ≤ 50% for the fixed-effect model and *I*^*2*^ > 50% for the random-effects model [21]. Effect sizes were compared using *z*-tests. A *P* value < 0.05 indicated statistically significant difference.

## Results

### Study selection

**Fig. 1:**
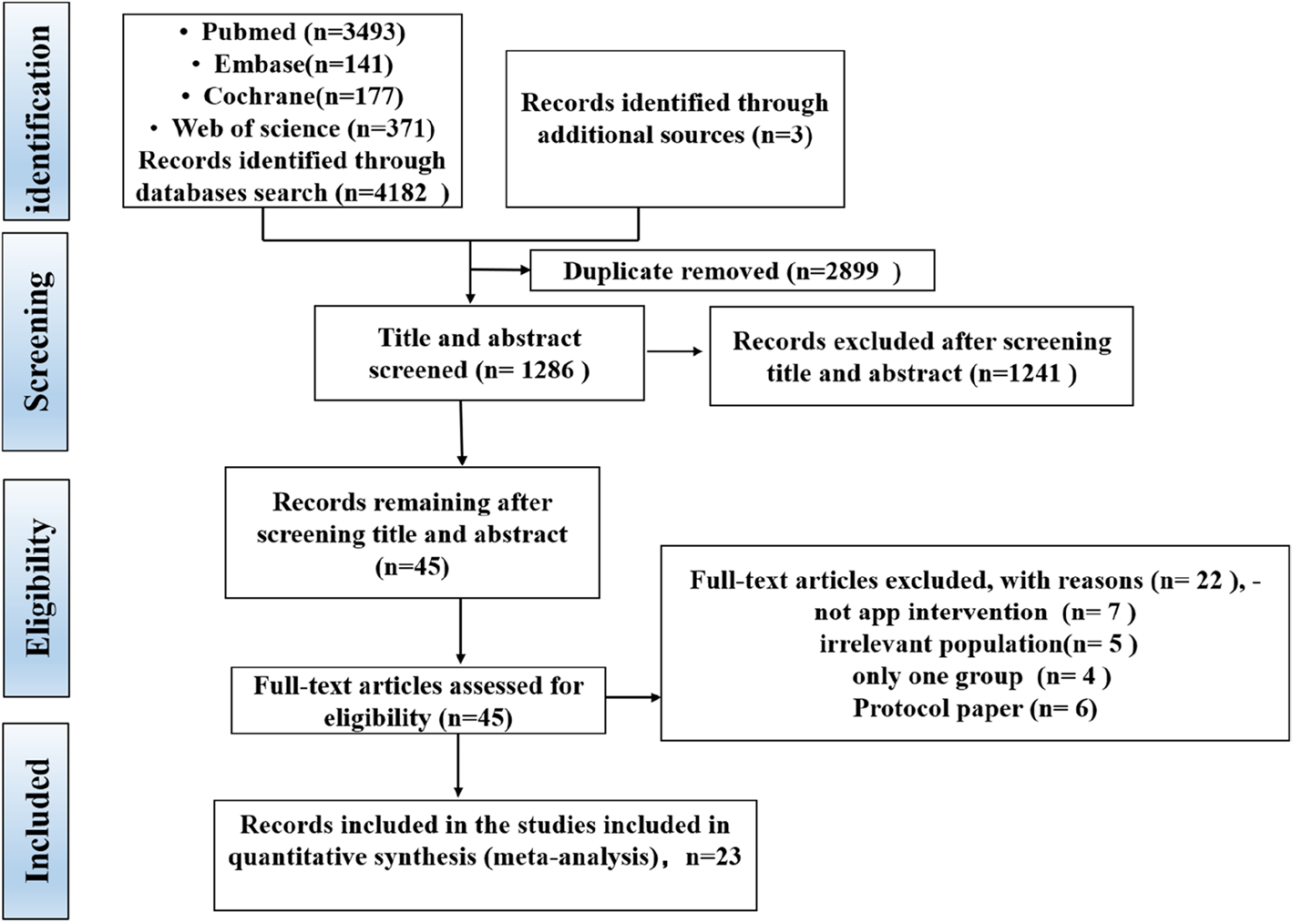
The PRISMA (Preferred Reporting Items for Systematic Reviews and Meta-Analyses) flow diagram

The screening procedure along with the criteria for excluding papers is shown in the PRISMA flow diagram (Fig. 1). The search retrieved 4185 citations, of which 2899 duplicates were removed. After exclusion of duplicates, a total of 1286 records were consequently assessed against the inclusion and exclusion criteria, and 45 full-text manuscripts were reviewed for eligibility. Of these 45 articles, 22 studies were excluded for several reasons. Therefore, a total of 23 records were included in this review.

### Quality of study

**Fig. 2:**
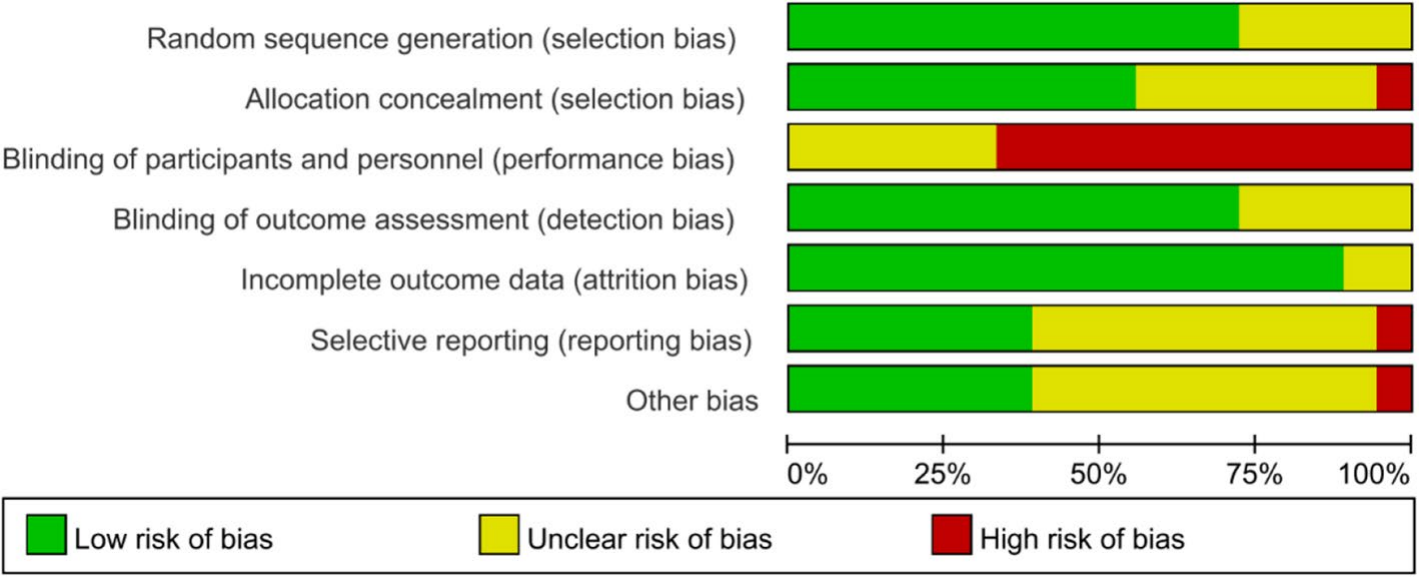
Risk of bias graph for RCTs: review authors’ judgments about each risk of bias item presented as percentages The x-axis represents the percentage of studies that were found to be of low (green), unclear (yellow), or high (red) risk of bias for each domain

**Fig. 3:**
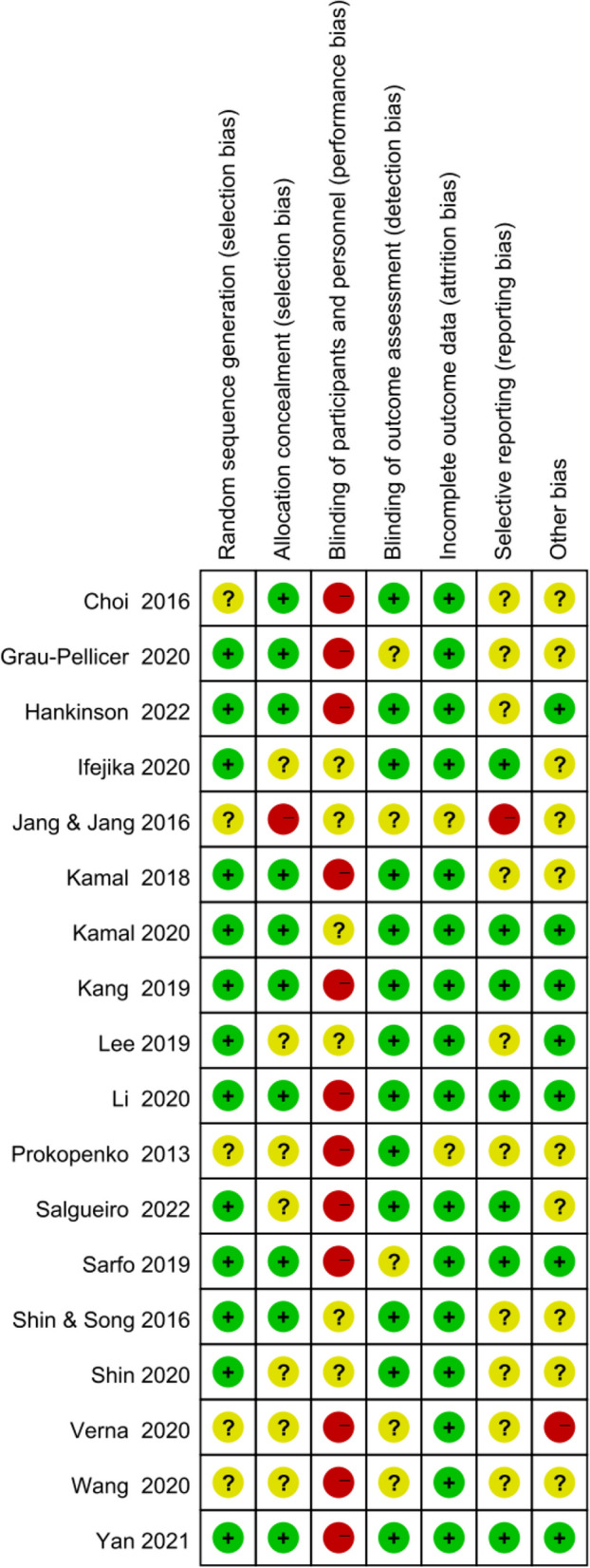
Risk of bias summary of RCTs

Figures 2 and 3 show the risk of bias judgment of RCTs. Thirteen studies [6, 11, 23–33] presented specific random sequence generation methods. Five trials [12, 34–37] that did not provide sufficient details about the randomisation method were rated as unclear with regard to random sequence generation. Allocation concealment was rated as low risk of bias in 10 trials [6, 12, 23–25, 27, 29, 31–33] (55.6%) and unclear in seven trials [11, 26, 28, 30, 35–37] (38.9%). Given the nature of mobile application interventions, blinding of study participants and health care personnel is not feasible, which inevitably causes performance bias. In total, 16 trials [6, 11, 12, 23–33, 35, 36] had a low risk of incomplete outcome data, whereas only seven trials [11, 25, 29–33] had a low risk of selective outcome reporting. The dropout and attrition rates were acceptable.

**Fig. 4:**
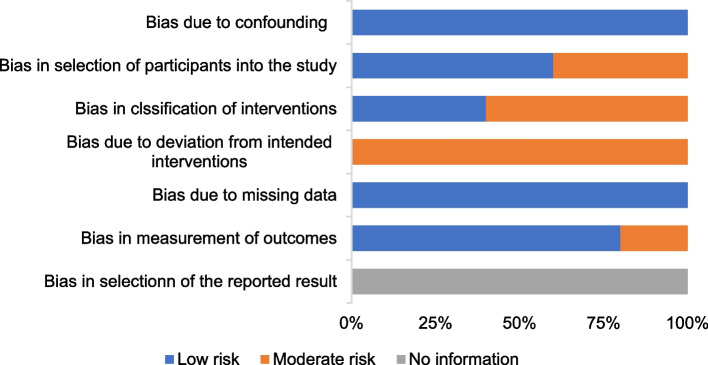
Risk of bias graph for CCTs: review authors’ judgments about each methodological quality item of ROBINS-I presented as percentages

Figures 4 shows the risk of bias of CCTs. Staggered recruitment time between the control and intervention groups resulted in little confounding bias. There was selection bias due to convenience sampling method used in 2 studies [38, 39]. Bias in the classifcation of interventions might be caused by lacking of random sampling and random grouping. There were bias due to deviations from intended interventions because the nature of mHealthl interventions. There were no significant sample loss and measurement bias. Generally, the quality of the included studies was acceptable.

### Characteristics of the included studies

Table 1 summarises the specific information extracted from the included studies.

**Table 1:** Special information of the studies included

Studies	Country	Study design	Study type	Participants, n	Age	Intervention duration	Intervention and control arm	Involvement of HCP	Measures to ensure app-based intervention adherence	Outcome
(Hankinson et al., 2022) [24]	Austria	Pilot study	RCT	E:11 C:11	Not reported	6-week	Intervention: the GotRhythm app Control: no app use, usual care	allied health professional	Not stated	FMA
(Sarfo et al., 2019) [30]	USA	Pilot study	RCT	E:30 C:30	The mean age was 55 (SD 13)	3 months	Intervention: Blue-toothed UA-767Plus BT BP device and a smartphone with an embedded app Control: SMS messages on healthy lifestyle behaviors	physicians and nurses	Not stated	BP medication adherence hypertension management competence self-regulation patient satisfaction side effects of antihypertensive medications stroke knowledge Barthel’s Index
(Fruhwirth et al., 2022) [40]	Austria	pilot study	CCT	E:21 C:21	E:41 ± 11 C:47 ± 8	3 months	Intervention: app Control: usual care without app	psychologists	Set and evaluate goals once a week	PA Self-reported nutrition alcohol consumption smoking behavior BMI BP
(Requena et al., 2019) [41]	Spain	pilot study	CCT	E:107 C:52	E: 57 ± 12 C: 59 ± 10	4-week	Intervention: Farmalarm app Control: usual care without app	occupational therapist	Not stated	BP diabetes mellitus HDL-C LDL-C smoking PA Stroke knowledge
(Kamal et al., 2020) [25]	Pakistan	Clinical outcome	RCT	E:141 C:137	E: 60.6 ± 12 C: 59.7 ± 14.3	6 months	Intervention: Movies4Stroke app Control: usual care without app	Not stated	constant SMS reminders	SBP DBP HbA1c LDL-C Mortality and the Number Needed to Treat Change in Functional Status
(Chae et al., 2020) [42]	Korea	Clinical outcome	CCT	E:17 C:6	E: 58.3 ± 9.3 C: 64.5 ± 9.6	3 months	Intervention: HBR apps Control: a printed handout to remind them about how to perform the four exercise tasks	Therapists	Weekly calls	WMT FMA-UE BDI ROM Grip power
(Lee, 2019) [27]	Korea	Clinical outcome	RCT	E:21 C:21	E: 61.67 ± 8.42 C: 64.24 ± 10.83	6 weeks	Intervention: virtual active app Control: usual care without app	Therapists	Not stated	mFRT FMA-LE
(Li et al., 2020) [34]	China	Clinical outcome	RCT	E:60 C:60	E: 58.3 ± 12.4 C: 60.8 ± 11.6	3 months	Intervention: WeChat app Control: telephone	Healthcare workers	Not stated	MBI
(Xu et al., 2021) [43]	China	Clinical outcome	CCT	E:51 C:50	E: 68.52 ± 3.15 C: 68.68 ± 3.18	3 months	Intervention: “Rehabilitation Guardian” App Control: health brochure	therapists	weekly calls	Hcy HDL-C TC FMA-LESE QoL patient satisfaction
(Yan et al., 2021) [32]	China	Clinical outcome	RCT	E:611 C:615	Among study participants, mean age was 65.7 [SD:8.2]	12 months	Intervention: SINEMA App Control: usual care without app	Village doctors	monthly follow-up visits	BP The “timed up and go” test PA QoL medication adherence
(Grau-Pellicer et al., 2020) [23]	Spain	Clinical outcome	RCT	E:24 C:17	E: 62.96 ± 11.87 C: 68.53 ± 11.53	8-week	Intervention: two mHealth apps, Fitlab® Training and Fitlab® Test Control: usual care without app	physical therapist	Not stated	PA Walking speed Walking endurance Functional mobility and risk of falling ADLs QoL patient satisfaction Barthel’s Index
(Kang et al., 2019) [33]	China	Clinical outcome	RCT	E:38 C:38	E: 50.47 ± 10.82 C: 52.33 ± 11.03	2-week	Intervention: SHEMA app Control: stroke health education booklet	trained research assistant	Not stated	stroke knowledge QoL
(Jang & Jang, 2016) [35]	Korea	Clinical outcome	RCT	E:10 C:11	E: 39.3 ± 18.1 C: 49.3 ± 14.0	4-week	Intervention: finger training app Control: usual care without app	Not stated	Not stated	MMT MFT PPT
(Paul et al., 2016) [39]	United Kingdom	pilot study	CCT	E:15 C:8	E: 56.3 ± 8.7 C: 55.3 ± 12.6	6-week	Intervention: STARFISH app Control: usual care without app	the researcher	At week three, each group of participants in the intervention arm attended the Clinical Research Facility to discuss their progress	PA BP FSS FMAT QoL PGWBI ADLs
(Prokopenko et al., 2013) [38]	Russia	Clinical outcome	RCT	E:24 C:19	The intervention group is represented by 24 patients at the age of 60–72 (Median = 61 [57;69]), control group-19 persons at the age of 60–72 (Median = 66 [61; 69])	14 days	Intervention: app Control: usual care without app	Healthcare workers	Not stated	MMSE MoCA
(Salgueiro et al., 2022) [31]	Spain	Clinical outcome	RCT	E:30 C:30	E: 57.27 ± 14.35 C: 64.53 ± 9.4	3 months	Intervention: Farmalarm app Control: conventional physiotherapy without app	main researcher	regularly calls	S-FIST S-PASS BBS
(Wang et al., 2020) [36]	China	Clinical outcome	RCT	E:150 C:150	E: 42.75 ± 0.16 C: 41.32 ± 2.16	12 months	Intervention: Ekangwang app Control: telephone	Not stated	Not stated	BP BMI LDL-C
(Kamal et al., 2018) [6]	Pakistan	Clinical outcome	RCT	E:99 C:98	E: 59.1 ± 11.6 C: 57.7 ± 11.1	3 months	Intervention: Talking Rx app Control: usual care without app	a study physician	Not stated	medication adherence
(Choi et al., 2016)[12]	Korea	Clinical outcome	RCT	E:12 C:12	E: 61.0 ± 15.2 C: 72.1 ± 9.9	2 weeks	Intervention: MoU-Rehab app Control: usual care without app	Not stated	Not stated	MMT FMAT QoL BDI
(Verna et al., 2020) [37]	Italy	Clinical outcome	RCT	E:12 C:12	E: 50.87 ± 12.37 C: 64.20 ± 10.94	4 weeks	Intervention: Te.M.P.O app with mismatches in the listened music theme sequences Control: Te.M.P.O app with no mismatches in the listened music theme sequences	Not stated	Not stated	MBI SSQoL
(Shin & Song, 2016) [28]	Korea	Clinical outcome	RCT	E:12 C:12	E:57.75 ± 14.03 C: 59.25 ± 9.75	4 weeks	Intervention: a SPVFTCT system Control: conventional rehabilitation program	Not stated	Not stated	trunk control ability mFRT eye open eye close
(Shin, 2020) [29]	Korea	Clinical outcome	RCT	E:12 C:12	Not stated	4 weeks	Intervention: a SPVFTCT system Control: conventional rehabilitation program	Not stated	Not stated	trunk control ability stride length step length
(Ifejika et al., 2020) [11]	USA	pilot study	RCT	E:17 C:19	The mean participant age was 54 years	6 months	Intervention: lose it app Control: pocket-sized CalorieKing Food & Exercise Journal	clinician	Not stated	Median weight change Depression

*E* Experimental, *C* Control, *RCT* Randomized controlled trial, *CCT* Clinical control trial, *HCP* Health care professional

*BP* Blood pressure, *FMA-LE* Fugl–Meyer Assessment, *BMI* Body Mass Index

*PA* Physical activity, *HDL-C* High-density lipoprotein cholesterol, *LDL-C* Low-density lipoprotein cholesterol, *SBP* Systolic Blood Pressure

*DBP* Diastolic Blood Pressure, *HbA1c* Glycosylated Hemoglobin A1c, *GPS* Global positioning system, *6MWT* 6-min walk test, *10MWT* 10-min walk test, *WMFT* Wolf Motor Function Test, *FMA-UE* Fugl-Meyer Assessment of Upper Extremity, *BDI* Beck Depression Inventory

*ROM* Range of motion, *mFRT* a modified functional reach test, *MBI* Modified Barthel Index, *Hcy* Homocysteine, *TC* Total cholesterol, *SE* Self-efcacy, *QoL* Health-related quality of life, *ADLs* Activities of daily living, *MMT* Manual Muscle Test, *MFT* Manual Function Test, *PPT* Purdue Pegboard Test, *MBI* Modified Barthel Index, *SSQoL* Stroke Specific Quality of Life scale

Twenty-three studies were published between 2013 and 2022 (82.6% in 2018 or later). All the included studies compared one app alone or app in conjunction with a package of participant support with a control arm. A total of 2983 participants were included, with sample sizes ranging from 21 [34] to 1226 [31]. Five studies [31–33, 35, 42]were conducted in China; four in Korea [12, 27, 28, 34]; three in Spain [23, 30, 43]; two in Austria [24, 41], USA [11, 29] and Pakistan [6, 25] and one in Russia [37], United Kingdom [38] and Italy [36]. Eighteen studies [6, 11, 12, 23–37] were RCTs, and five [38, 39, 41–43] were CCTs. Seventeen articles [6, 12, 23, 25–28, 30–35, 37, 39, 42] presented clinical outcomes, whereas six articles[11, 24, 29, 38, 41, 43] were pilot studies. Regarding the intervention duration of the studies, eight lasted ≤ 1 month [12, 27, 28, 32, 34, 36, 37, 43], four [23, 24, 26, 38] lasted 1–3 months, and eleven [6, 11, 25, 29–31, 33, 35, 39, 41, 42] lasted ≥ 3 months. The participants in the studies were primarily young and middle-aged stroke survivors, and only four studies primarily involved patients 60 years or older [12, 23, 26, 42]. More than half of the studies had a sample of less than 50 participants.

The control group received usual care without the use of the app in 14 trials [6, 12, 24–28, 30, 31, 34, 37, 38, 41, 43], SMS messages in one trial [29], a printed handout [39] or health brochure [32, 42] or pocket-sized material [11] in four trials, telephone follow-up in two trials [33, 35] and the same app compared with the intervention group but with different functionality in one trial [36].

### Involvement of HCPs and measures to ensure app-based intervention adherence

Sixteen trials [6, 11, 23, 24, 26, 29–33, 37–39, 41–43] involved HCPs in app use, and the remaining seven trials [12, 25, 27, 28, 34–36] did not specify information of HCPs involved in app use (Table 1). The involvement of HCPs varied; most of the trials involved HCPs to prescribe patient’s rehabilitation training [24, 33, 38], guide the training [23, 26, 30, 37, 39], instruct patients on how to use the app or measure blood pressure using the BP device and remind patients to use the app [6, 29–32, 41–43].

Only seven trials reported measures to ensure app-based intervention adherence; the measurements include setting and evaluating goals once a week [41], sending constant SMS reminders [25] performing regular phone calls [30, 39, 42] and conducting follow-up visit [31, 38].

### App characteristics

A total of 18 different mobile applications were used across the studies, and four did not provide respective information of the app. The functionality of the apps varied across different trials. Mobile applications were used for three target areas amongst stroke survivors: three in education [25, 31, 32], nine in self-care [6, 11, 23, 29, 35, 38, 41–43] and 11 in rehabilitation [12, 24, 26–28, 30, 33, 34, 36, 37, 39].

With regard to education, one app delivered 5-min videos on various stroke-related topics, which was developed by biomedical and software engineers of Aga Khan Development Network Electronic Health (eHealth) Resource Center in collaboration with stroke specialists, rehabilitation and swallowing experts and epidemiologists [25]. One app delivered a tailored motivational SMS text message [31], and one app has a stroke health-education content covering 12 topics of risk factors in patients with stroke (e.g. stroke history and hypertension), which can be browsed by participants for several times without time and location limitation [32]. With regard to self-care, the majority of apps have a self-monitoring function or medication reminder, health information, assessment, feedback, health service and social support. As for rehabilitation, apps can detect a variety of physical activities and transmit rehabilitation-related data to the server computer, which are shared with the therapist. Most of these apps need other devices to achieve these functions. In addition, some apps help patients to obtain access to visual and auditory feedback on their excise by viewing the display on the screen of synchronous equipment. App characteristics are outlined in Table 2.

**Table 2:** Special information on app characteristics

First author, publication year	APP name	APP development purpose		Devices used with the app and functionality	Main feature of the app
		**Generally objective**	**Special objective**		
(Hankinson et al., 2022) [24]	GotRhythm	rehabilitation	To deliver individualized music-motor therapy to stroke survivors and provide feedback on motor performance	non-intrusive sensors (wireless inertial motion units [IMU]; Mbientlab Inc., San Francisco, CA): transmit acceleration, gyroscope and magnetometer data to the GotRhythm app	detect a variety of physical activities converts the raw sensor data into attitude angles (yaw, pitch and roll) in real time
(Sarfo et al., 2019) [30]	Name not specified	self-care	improve BP control	Blue-toothed UA-767Plus BT BP device	monitoring and reporting BP measurements
(Fruhwirth et al., 2022) [40]	PRESTRO	self-care	combining motivational support for a healthy lifestyle, medication adherence, and stroke education	No device	conveys key facts about stroke provides motivational support for a healthy lifestyle, and a reminder function for medication intake and blood pressure measurement
(Requena et al., 2019) [41]	Farmalarm	self-care	to increase stroke awareness and treatment compliance through visual and audible alerts	No device	Medication reminding blood pressure /capillary glycaemia monitoring and sharing Establish contact
(Kamal et al., 2020) [25]	Movies4Stroke	education	Deliver poststroke education to stroke survivor and caregiver dyads returning to the community	No device	5-min videos on various stroke-related topics: different skills such as swallowing exercises, different rehabilitation exercises, and nasogastric tube feeding emergency preparedness medications secondary stroke prevention
(Chae et al., 2020) [42]	smartwatch	rehabilitation	detection of home exercise activities	off-the-shelf smartwatch	The smartphone app for patients acts as a platform for starting the smartwatch, detecting home rehabilitation, and transmitting exercise time data to the server computer and share with the therapist
(Lee, 2019) [27]	Virtual Active	rehabilitation	To improve lower limb motor function, trunk sitting balance, and gait	posturography system (GB300; Metitur Ltd., Jyvaskyla, Finland) a stationary bike (MOTOmed viva, RECK-Technik, Betzenweiler, Germany), projector (BX327, LG, Seoul, South Korea)	motion tracking provides shooting videos of mountains, valleys, and cities recognize three virtual landmarks of the head and shoulders and draws a movement pattern
(Li et al., 2020) [34]	WeChat	rehabilitation	assess the post discharge functional status of patients following a stroke	No device	including the videoconference and telephone call functions
(Xu et al., 2021) [43]	Rehabilitation Guardian	Self-care	Improve self-efficacy, quality of life, and motor function of stroke patients	No device	Patient diary Health information Consultation Health reminder
(Yan et al., 2021) [32]	SINEMA	Education	a voice message–based intervention package for promoting secondary prevention	third-party dispatching platform and a message bank	a voice of stroke education messages system for patients patients’ profiles follow-up visits training performance indicators follow-up visits reminders
(Grau-Pellicer et al., 2020) [23]	Fitlab®	Self-care	promote adherence to physical activity	A pedometer (model UW-100, UW-101® A&D®)	1) to supervise adherence to PA; 2) to assess mood, effort, recovery, wellness and fatigue questionnaires; 3) to have bidirectional feedback
(Kang et al., 2019) [33]	SHEMA	Education	To improve knowledge of stroke risk factors	No device	The content covered the 12 health- education topics
(Jang & Jang, 2016) [35]	a finger training application	rehabilitation	finger training	A tablet PC	registration section evaluation section, when the patient placed the affected fingers on the screen, the positions of five fingertips were measured The training section consisted of five programs (stretching, flexion, extension, opposition, and thumb abduction) and the patient could choose one of the five programs
(Paul et al., 2016) [39]	STARFISH	Self-care	Increasing PA	No device	feedback self-monitoring social support
(Prokopenko et al., 2013) [38]	Name not specified	rehabilitation	cognitive neurorehabilitation	computerized Schulte's tables	Four aspects of attention training (sustained, selective, divided, and alternating ones)
(Salgueiro et al., 2022) [31]	Farmalarm	rehabilitation	therapeutic exercises performed by an App on trunk control, balance, and gait	No device	Medication reminding blood pressure /capillary glycaemia monitoring and sharing Establish contact
(Wang et al., 2020) [36]	the Ekangwang app	Self-care	managing BP and improving the self-care ability	the Bluetooth sphygmomanometer Yuwell 680A, the Bluetooth blood glucose meter LipidEx3B, and a wearable bracelet	monitoring service (blood pressure data, blood pressure early warning, and intelligent bracelet) health service (health knowledge, exercise plan, diet plan, and medication reminder), hospital service (doctor’s advice in the hospital, test report, and my case) doctor service (remote guidance and online consultation)
(Kamal et al., 2018) [6]	Talking Rx	Self-care	boost medication adherence and medication health literacy	No device	1) Daily Interactive Voice Response (IVR) call services providing information about specific statin and antiplatelet, 2) Daily tailored medication reminders for patients to take their statin and antiplatelet medication and 3) Weekly Life Style Modification Messages that engaged the patient and were also related to medication adherence
(Choi et al., 2016) [12]	MoU-Rehab	rehabilitation	improve upper limb function	a tablet PC (Galaxy Note 10.0, Samsung, Seoul, South Korea) and a smartphone (Galaxy S2, Samsung) with a Bluetooth connection	The MoU-Rehab consisted of mobile game applications. All game applications were designed to improve strength, endurance, range of motion, control, speed, and accuracy of movement in the upper extremity
(Verna et al., 2020) [37]	Temporal Musical Patterns Organisation (Te.M.P.O)	rehabilitation	neurological rehabilitation	No device	allowed to exercise the cognitive functions using sudden variation of music tone during the listening of a sequence of different music themes
(Shin & Song, 2016) [28]	Name not specified	rehabilitation	Trunk control training	a smartphone (iPhone 5, Apple, USA, 2013) inserted balance board (Pedalo balance top)	recognize the changes in trunk movements
(Shin, 2020) [29]	Name not specified	rehabilitation	Trunk control training	a smartphone (iPhone 5, Apple, USA, 2013) inserted balance board (Pedalo balance top)	recognize the changes in trunk movements
(Ifejika et al., 2020) [11]	Lose it	Self-care	Lose weight	No device	Search for foods, including restaurant items, or scan the bar codes of grocery items, with direct upload onto the Lose It! platform

### Intervention effectiveness

#### Medication adherence

**Fig. 5:**
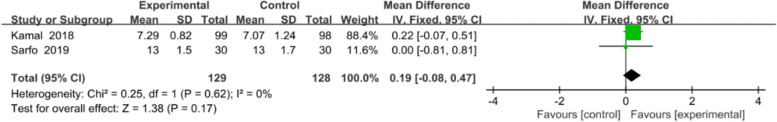
Meta-analysis results and forest plot of the effect of app-based interventions on medication adherence

Two studies with a total of 257 patients were included in the meta-analysis. The two studies used 8-item Morisky Medication Adherence Scale 8 to assess medication adherence. Inverse-variance-weighted linear meta-analysis of MD (Hedge’s g) on these studies revealed a medium effect size of 0.19 favouring mobile application, but MD was not significant (0.19, 95% CI [− 0.08, 0.47];* P* = 0.17; Fig. 5).

### Functional outcomes

**Fig. 6:**
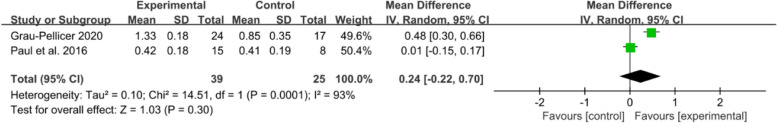
Meta-analysis results and forest plot of the effect of app-based interventions on 10 MWT

An array of functional outcomes was measured across the trials, which included a 10-min walk test (10 MWT), Barthel index, Fugl–Meyer assessment (FMA-LE), trunk control ability and Fugl–Meyer assessment of the upper extremity (FMA-UE). Two studies with a total sample size of 64 subjects were included in meta-analysis to assess the effect of mobile application on 10 MWT. Meta-analysis for 10 MWT (Fig. 6) demonstrating a non-significant effect in favour of the app intervention (MD 0.24, 95% CI [− 0.22 to 0.70]; *P* = 0.30), with a high statistical heterogeneity (I^2^ = 93%).

**Fig. 7:**
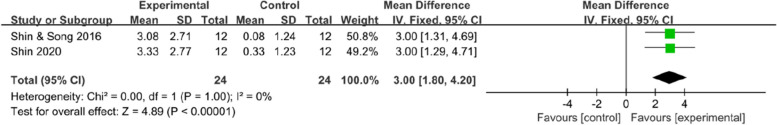
Meta -analysis results and forest plot of the effect of app-based interventions on trunk control

Data on trunk control ability were available in two trials (48 patients), which all used the trunk impairment scale. The results indicated that mobile application interventions could improve the trunk control ability of stroke survivors (MD 3, 95% CI [1.80 to 4.2]; *P* < 0.00001), with no statistical heterogeneity (I^2^ = 0%, Fig. 7).

**Fig. 8:**
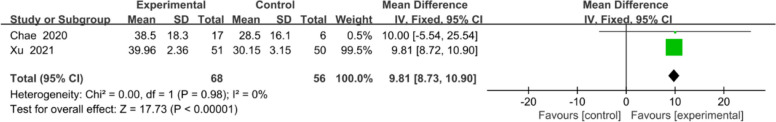
Meta-analysis results and forest plot of the effect of app-based interventions on FMA-UE

The overall effect (Fig. 8) revealed that mobile application-based intervention could effectively improve FMA-UE, and the forest plot showed no heterogeneity amongst studies (MD 9.81, 95% CI [8.72 to 10.90]; *P* <0.00001), with no statistical heterogeneity (I^2^ = 0%).

**Fig. 9:**
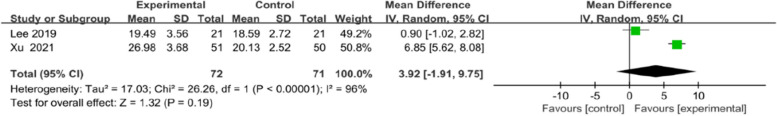
Meta-analysis results and forest plot of the effect of app-based interventions on FMA-LE

**Fig. 10:**
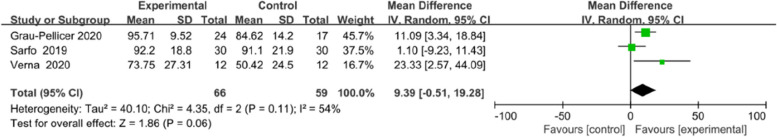
Meta-analysis results and forest plot of the effect of app-based interventions on Barthel index

Meta-analysis for FMA-LE and Barthel index all favoured the use of an app, but no statistical differences in FMA-LE (MD 3.92, 95% CI [1.91to 9.75]; *P* = 0.19; Fig. 9) or Barthel index (MD 9.39, 95% CI [−0.51 to 19.28]; *P* = 0.06; Fig. 10) were observed between the intervention and control groups.

### Cardiovascular risk factor

**Fig. 11:**
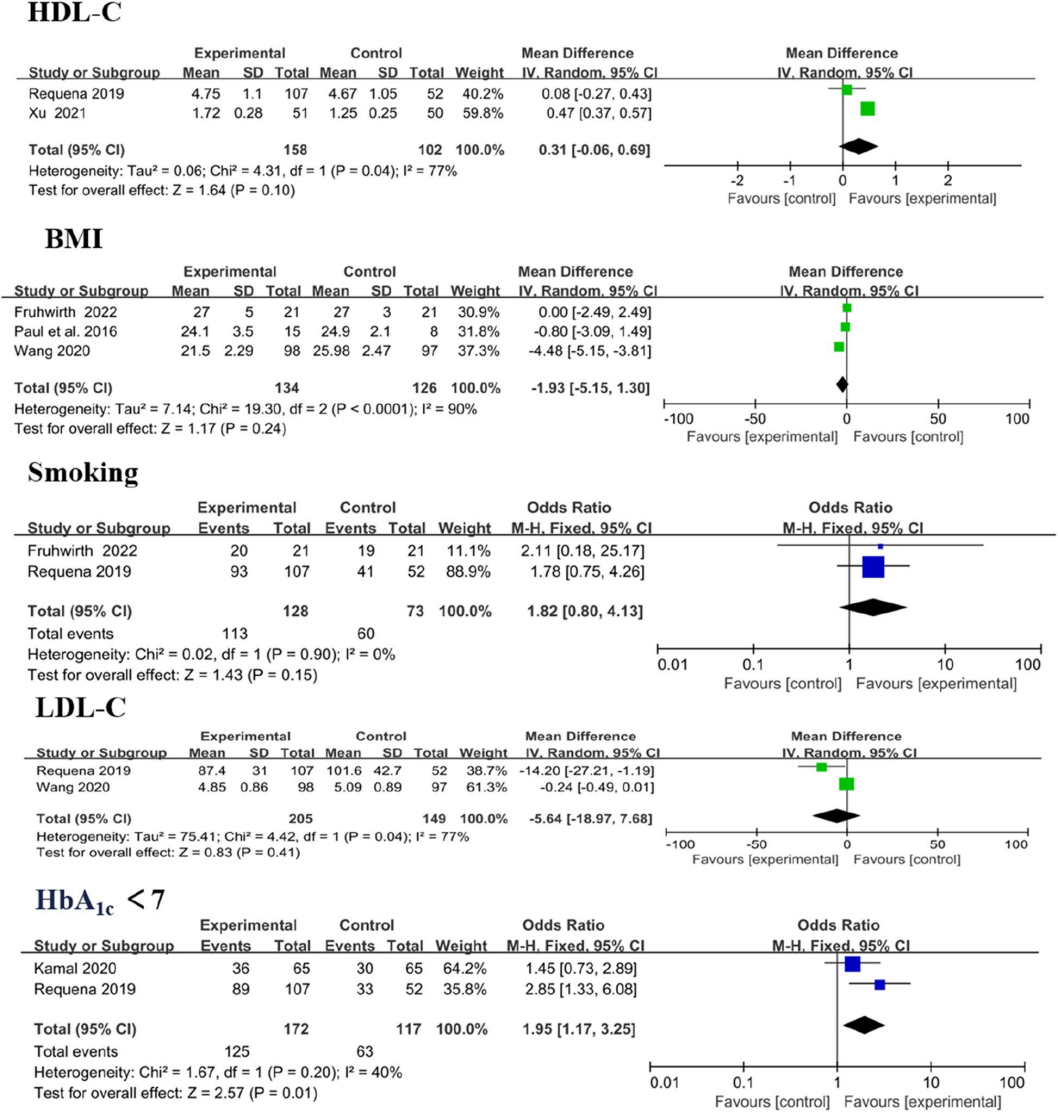
Forest plots of HDL-C, BMI, smoking, LDL-C and HbA_1c_ < 7 results

Cardiovascular risk factors included systolic blood pressure (SBP), diastolic blood pressure (DBP), high-density lipoprotein cholesterol (HDL-C), low-density lipoprotein cholesterol (LDL-C), body mass index (BMI), smoking and glycosylated haemoglobin A_1c_ (HbA_1c_). Analysis showed significant differences in LDL-C (MD − 0.33, 95% CI [− 0.54 to − 0.11]; *P* = 0.003) and HbA_1c_ < 7 levels (MD 1.95, 95% CI [1.17 to 3.25]; *P* = 0.01). Amongst the outcomes that were reported by more than one study, no significant difference in modifying HDL-C (MD 0.31, 95% CI [− 0.06 to 0.96]; *P* = 0.10), BMI (MD − 1.93, 95% CI [− 5.15 to 1.30]; *P* = 0.24) and smoking (MD 1.82, 95% CI [0.80 to 4.13];* P* = 0.15; Fig. 11) was observed between the intervention and control group.

**Fig. 12:**
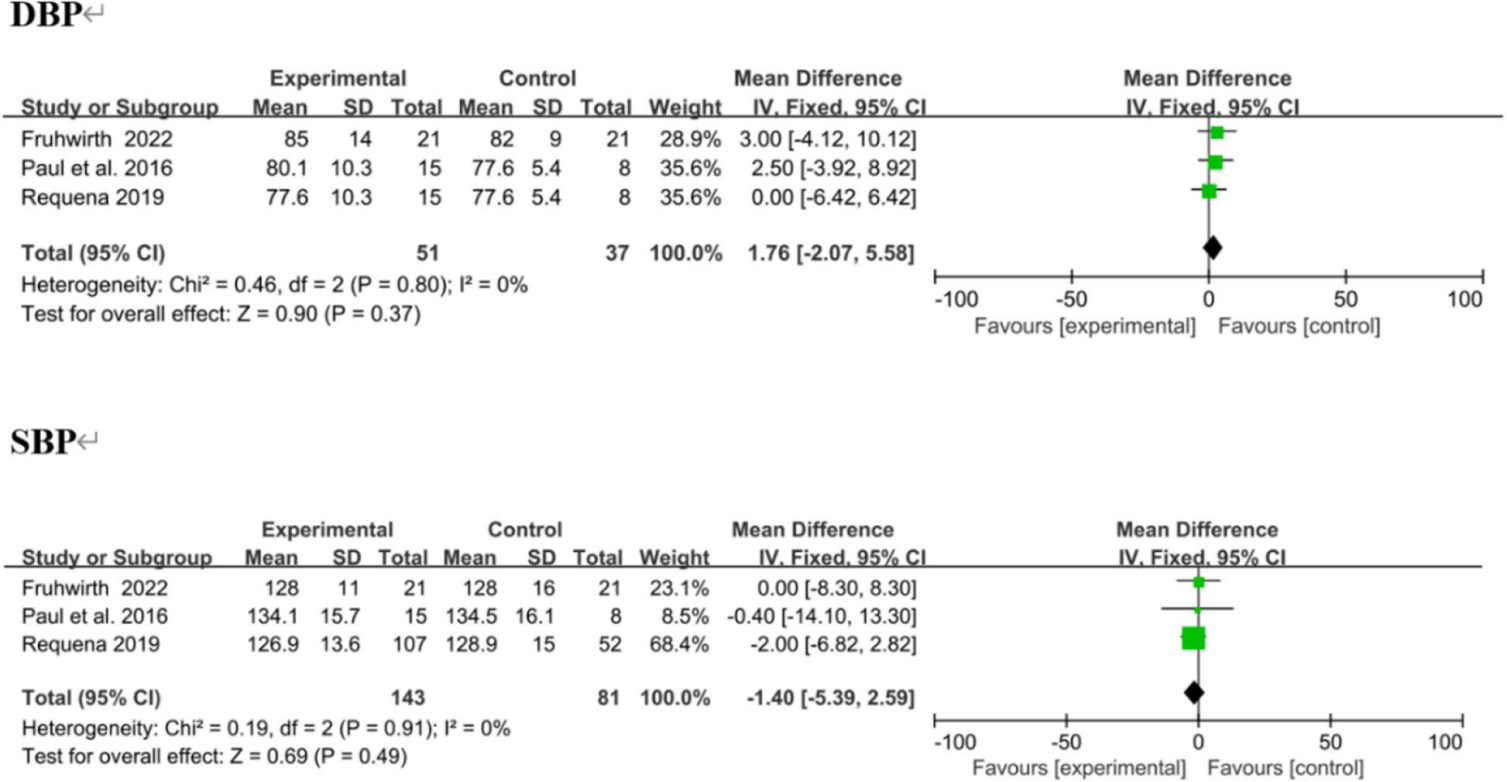
Forest plots of DBP and SBP results. Negative MDs between the two groups favour the mobile application-based intervention

Three trials reported SBP and DBP as outcomes, [38, 41, 43] with no significant change. In overall effect analysis, no significant differences in DBP (MD 1.76, 95% CI [− 2.07 to 5.58]; *P* = 0.37) or SBP (MD − 1.40, 95% CI [− 5.39 to 2.59]; *P* = 0.49) were observed between the two groups (Fig. 12).

### Quality of life (QoL)

**Fig. 13:**
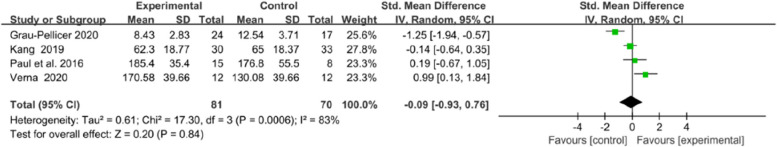
Forest plots of Qol results

Qol was evaluated in four trials, using the European Quality of Life–Five Dimensions (EQ-5D) and the Stroke Specific Quality of Life Scale (SS-QOL). The integrated results showed no significant difference in QoL between the two groups (MD =  − 0.09, 95% CI =  − 0.93 to 0.76, *P* = 0.84; I^2^ = 83%, *P* = 0.0006; Fig. 13).

### Knowledge of stroke

**Fig. 14:**
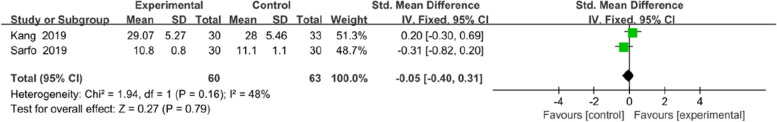
Forest plots of knowledge of stroke results

Two trials assessed the effectiveness of the mobile application on knowledge of stroke. The tools used to assessed knowledge on stroke were the14-item hypertension and stroke knowledge questionnaire score [29] and stroke-knowledge questionnaire [32]. Meta analysis for these two studies of 123 participants (intervention n = 60, control *n* = 63) showed that mobile application interventions did not exert a statistically significant effect on knowledge of stroke (MD =  − 0.05, 95% CI =  − 0.40 to 0.31, *P* = 0.79; Fig. 14).

## Discussion

### Principal findings

In this systematic review and meta-analysis, evidence was synthesised from 18 RCTs and five CCTs (n = 2983 patients) that assessed the effectiveness of mobile application interventions in patients with stroke. Mobile applications targeting stroke survivors primarily encompassed three areas: rehabilitation, education and self-care. Regarding the involvement of HCPs and measures to ensure app-based intervention adherence, most of the trials involved HCPs in app use, but a few trials provided details about measures to ensure app adherence during intervention. The participants in the studies included young and middle-aged adults. The results showed that on average, mobile application intervention had a statistically significant improvement in trunk control ability, FMA-UE, LDL-C and HbA_1c_ < 7, but little to no effect was observed on medication adherence, 10 MWT, Barthel index, SBP, DBP, HDL-C, BMI, smoking, Qol and knowledge of stroke compared with the control group. The evidence was modest; however, this finding, should be cautiously interpreted because of the app features, content diversity, clinical and methodological heterogeneity amongst studies and small sample sizes.

The total effect indicated that the trunk control ability of the mobile application intervention group was better than that of the control group. Using mobile applications, a visual feedback training environment can be built to help trunk control and gait of patients with stroke, which helps the user focus on the task [27, 28]. However, in generalising the effects of mobile applications on trunk performance in patients with stroke, further studies with a larger number of subjects, greater homogeneity with regard to the device used and equal total training times for the mobile application group and control group must be conducted [27, 28].

Statistical analysis carried out in the present review revealed favourable results of mobile application intervention on FMA-UE amongst stroke survivors, which is consistent with previous studies [40]. These effects may be due to the advantages inherent to mobile application interventions, including timely feedback, overcoming the barriers of distance, travel time and personalisation, which enables patients to receive continuous rehabilitation in a feasible and convenient manner. Hao et al. [44] highlights that the continuation of rehabilitation promotes the recovery of functional deficits, resulting in optimal outcomes amongst stroke survivors.

This meta-analysis showed that the use of mobile application interventions was associated with significant improvements in HbA_1c_ < 7. The findings of the current study are consistent with those of Liu et al. [45] who observed the use of mobile app-assisted self-care interventions, which led to an SMD of − 0.44 and an absolute MD of − 0.49% with regard to HbA_1c_ level [45]. Using mobile application, patients’ engagement in the behaviour of monitoring blood glucose was further developed, which helped control HbA_1c_. [45]In addition, some app creators incorporate a feedback module into the design of the application, and feedback will improve lifestyle choices, thereby decreasing HbA1c [46].

Integrated results showed that mobile application interventions could effectively improve LDL-C amongst stroke survivors. This finding is consistent with that of Xu et al. [42], that is, mobile health can reduce the LDL-C level significantly. These results are due to mobile applications, which can facilitate the remote management of health issues and data, patient–care provider communication, provision of personalised self-care recommendations and decision-making. Moreover, evidence regarding the effects of mobile application interventions on the LDL-C level warrants further examination.

### Relationship with previous published literature

Previous systematic reviews have assessed the effectiveness of mobile applications in medication adherence [47], lifestyle modification in type 2 diabetes [48], perinatal depression and anxiety [49] and nutritional outcomes in adults with chronic diseases. Many reviews have reported that mobile applications are effective tools, and that their use results in positive effects. To the best of our knowledge, four other systematic reviews have been published on this topic amongst stroke survivors [16–19]. However, two previous reviews did not conduct a systematic review that accompanying meta-analysis [16, 17]. Furthermore, one of them was limited to the efficacy of rehabilitation amongst stroke survivors [13]; the other addressed the role of mHealth apps supporting self-management of health and function amongst survivors [14]. In addition, although two other recent systematic review and meta-analysis aimed at assessing the effects of mobile applications in patients with stroke [15, 16], a mobile technology-based intervention was included (e.g. personal digital assistants), and it does not focus solely on mobile application. Therefore, the present systematic review is unique, and it goes beyond the findings of previous reviews that focused only on mobile application, including evidence from newly published studies.

### Overall completeness and applicability of the evidence

Generalisability and applicability of our results may be more or less affected when considering the following factors. Firstly, similar to other study [50], older people were underrepresented in the included trials, although stroke highly affected older adults, which may reduce the usefulness of the information provided by trials on efficacy. In addition, the magnitude of all included studies was performed in Asian countries, and the results of this study may not be generalised to a wider population. Secondly, almost half of the included trials involved the use of simple mHealth apps. Further research must be conducted to determine feasibility, efficacy and acceptance of the wearable devices interlock with the mobile application. Wearables are widely used, and they have shown promising results in the field of healthcare because of their ability of deformability and compliance [51]. Furthermore, multimodal Biofeedback rehabilitation may represent a good alternative for post-stroke patients and be a suitable adjunct to physical therapy [52]. A systematic review and meta-analysis of literature comparing traditional rehabilitation therapy and traditional plus VR rehabilitation therapy found that immersive virtual reality rehabilitation treatment may further improve rehabilitation outcomes and counld become a new option for rehabilitation after stroke in the future [53]. Augmented Reality (AR) technology for rehabilitation after stroke is in its infancy and warrants further investigation [54].Considering the sample size, app content, duration of the interventions, care settings and intervention characteristics, the included studies were diverse clinically and methodologically. However, the mechanism by which these clinical, methodological and contextual differences might affect the results remains unclear, although no statistically significant heterogeneity is observed amongst the included studies. Given the complex nature of mobile application interventions, their efficacy was directly associated with a range of contextual factors [55].

### Limitations

This study has its own limitations that are worth considering. Firstly, language biases might exist because these searches were conducted in English, which may limit the cross-cultural generalisability of our findings. Secondly, relying primarily on randomised controlled studies is difficult because of the nature of the available evidence. However, the inclusion of quasi-experimental studies allows us to address outcomes from mobile application interventions that have not been sufficiently studied in randomised controlled trials and justified. In general, the inclusion of quasi-experimental studies is justified when more rigorous trials are lacking [56]. Thirdly, scopus database has not been reviewed and we indeed should have reviewed scopus database in future studies. However, we searched PubMed, EMBASE, Cochrane and Web of Science databases. The keyword search with PubMed offers optimal update frequency and includes online early articles; other databases can rate articles by number of citations, as an index of importance. For citation analysis, Scopus offers about 20% more coverage than Web of Science, whereas Google Scholar offers results of inconsistent accuracy. PubMed remains an optimal tool in biomedical electronic research. Scopus covers a wider journal range, of help both in keyword searching and citation analysis, but it is currently limited to recent articles (published after 1995) compared with Web of Science. Google Scholar [57]. In addition, given the small amount of studies, subgroup analyses were not examined, and publication bias was not explored. Finally, differences in the number of participants, methods, intervention contents, frequency, measurements and follow-up time resulted in heterogeneity.

### Implications for clinical practice and research

Mobile apps may be a promising tool to complement routine clinical care amongst stroke survivors. However, the implementation of mobile applications amongst stroke survivors is still in its infancy. Additional research that examines the effects of interventions is necessary. In addition, clarifying whether the present limited efficacy holds true and identifying in which circumstances their potential could be increased are potentially relevant fields that should be investigated systematically. Furthermore, given the increase in aging population, further studies that will involve older stroke survivors, who are the largest potential user population, must be conducted. Older adults must use digital health tools and mobile health applications to help them in independent living and self-management of (chronic) illnesses [58]. Smart phone ownership amongst adults aged 65 and older has increased substantially. As of 2017, around four in 10 (42%) adults aged 65 years and older were using a smartphone [59]. Based on the European Union commission’s 2012–2020 eHealth Action Plan, current mHealth landscape lacks user-friendly tools and services for older patients [58]. Therefore, understanding the needs of older stroke survivors is important to design, develop and evaluate the mobile application intervention amongst this population. Finally, Weisel et al. [55] highlighted that engagement is linked to the efficacy of apps, and adherence to app should be further investigated.

## Conclusions

Our review found that mobile applications can potentially facilitate the trunk control ability, Fugl–Meyer assessment of upper extremity, low-density lipoprotein cholesterol and glycosylated haemoglobin A1c (HbA1c) < 7 levels. However, patients assigned to the mobile application group and the conventional care group did not difer signifcantly in medication adherence, 10-min walk test (10 MWT), Barthel index, systolic blood pressure, diastolic blood pressure, high-density lipoprotein cholesterol, body mass index, smoking, health-related quality of life and knowledge of stroke. In addition, generalisable evidence to unreservedly recommend the use of mobile applications amongst stroke survivors as a substitute to conventional management is still lacking because of the clinical and methodological heterogeneity amongst studies, small sample sizes and disparity in app features, content and follow-up. Given the growing popularity of mobile applications worldwide and in order for mHealth approaches to be widely embraced, more studies using rigorous designs, with long-term follow-up and representative samples of older adults are warranted to understand the sustainability of mobile application intervention effects.

### Supplementary Information

**Additional file 1.**

**Additional file 2.**

## Data Availability

The original contributions presented in the study are included in the article, further inquiries can be directed to the corresponding author on reasonable request.
