# Supplementary material for: Effectiveness of mobile application interventions for stroke survivors: systematic review and meta-analysis
Source: BMC Med Inform Decis Mak. 2024 Jan 2;24:6. doi: 10.1186/s12911-023-02391-1 (PMC10763083; doi:10.1186/s12911-023-02391-1)
Supplement: Supplementary file 2 — Additional file 2. [file 12911_2023_2391_MOESM2_ESM.docx]

**Detailed search strategy for each database**

# Pubmed

 (((("Stroke"[Mesh]) OR ((((((((((((((((((((((((((((Strokes[Title/Abstract]) OR (Cerebrovascular Accident[Title/Abstract])) OR (Cerebrovascular Accidents[Title/Abstract])) OR (CVA (Cerebrovascular Accident[Title/Abstract]))) OR (CVAs (Cerebrovascular Accident[Title/Abstract]))) OR (Cerebrovascular Apoplexy[Title/Abstract])) OR (Apoplexy, Cerebrovascular[Title/Abstract])) OR (Vascular Accident, Brain[Title/Abstract])) OR (Brain Vascular Accident[Title/Abstract])) OR (Brain Vascular Accidents[Title/Abstract])) OR (Vascular Accidents, Brain[Title/Abstract])) OR (Cerebrovascular Stroke[Title/Abstract])) OR (Cerebrovascular Strokes[Title/Abstract])) OR (Stroke, Cerebrovascular[Title/Abstract])) OR (Strokes, Cerebrovascular[Title/Abstract])) OR (Apoplexy[Title/Abstract])) OR (Cerebral Stroke[Title/Abstract])) OR (Cerebral Strokes[Title/Abstract])) OR (Stroke, Cerebral[Title/Abstract])) OR (Strokes, Cerebral[Title/Abstract])) OR (Stroke, Acute[Title/Abstract])) OR (Acute Stroke[Title/Abstract])) OR (Acute Strokes[Title/Abstract])) OR (Strokes, Acute[Title/Abstract])) OR (Cerebrovascular Accident, Acute[Title/Abstract])) OR (Acute Cerebrovascular Accident[Title/Abstract])) OR (Acute Cerebrovascular Accidents[Title/Abstract])) OR (Cerebrovascular Accidents, Acute[Title/Abstract]))) AND ("Mobile Applications"[Mesh])) OR ((((((((((((((((((((((((((((Application, Mobile[Title/Abstract]) ) OR (Applications, Mobile[Title/Abstract])) OR (Mobile Application[Title/Abstract])) OR (Mobile Apps[Title/Abstract])) OR (App, Mobile[Title/Abstract])) OR (Apps, Mobile[Title/Abstract])) OR (Mobile App[Title/Abstract])) OR (Portable Software Apps[Title/Abstract])) OR (App-based [Title/Abstract])) OR (App, Portable Software[Title/Abstract])) OR (Portable Software App[Title/Abstract])) OR (Software App, Portable[Title/Abstract])) OR (Portable Software Applications[Title/Abstract])) OR (Application, Portable Software[Title/Abstract])) OR (Portable Software Application[Title/Abstract])) OR (Software Application, Portable[Title/Abstract])) OR (Smartphone Apps[Title/Abstract])) OR (App, Smartphone[Title/Abstract])) OR (Apps, Smartphone[Title/Abstract])) OR (Smartphone App[Title/Abstract])) OR (Portable Electronic Apps[Title/Abstract])) OR (App, Portable Electronic[Title/Abstract])) OR (Electronic App, Portable[Title/Abstract])) OR (Portable Electronic App[Title/Abstract])) OR (Portable Electronic Applications[Title/Abstract])) OR (Application, Portable Electronic[Title/Abstract])) OR (Electronic Application, Portable[Title/Abstract])) OR (Portable Electronic Application[Title/Abstract]))) AND ("randomized controlled trial" or "RCT" or "quasi-experiment" or "trial" or "intervention" or “randomized clinical trial” or “controlled clinical trial” )

# Web of science

TS=(Stroke OR Strokes OR (Cerebrovascular Accident) OR (Cerebrovascular Accidents) OR (CVA (Cerebrovascular Accident)) OR (CVAs (Cerebrovascular Accident)) OR (Cerebrovascular Apoplexy) OR (Apoplexy, Cerebrovascular) OR (Vascular Accident, Brain) OR (Brain Vascular Accident) OR (Brain Vascular Accidents) OR (Vascular Accidents, Brain) OR (Cerebrovascular Stroke) OR (Cerebrovascular Strokes OR Stroke), Cerebrovascular OR (Strokes, Cerebrovascular) OR Apoplexy OR (Cerebral Stroke) OR (Cerebral Strokes) OR (Stroke, Cerebral) OR (Strokes, Cerebral) OR (Stroke, Acute) OR ( Acute Stroke) OR (Acute Strokes) OR (Strokes, Acute) OR (Cerebrovascular Accident, Acute) OR (Acute Cerebrovascular Accident) OR (Acute Cerebrovascular Accidents) OR (Cerebrovascular Accidents, Acute)) AND TS=(Mobile Applications OR (Application, Mobile) OR (Applications, Mobile) OR (Mobile Application) OR (Mobile Apps) OR (App, Mobile) OR (Apps, Mobile) OR (Mobile App) OR (Portable Software Apps) OR (App, Portable Software) OR (Portable Software App) OR (Software App, Portable) OR (Portable Software Applications) OR (Application, Portable Software) OR (Portable Software Application) OR (Software Application, Portable) OR (Smartphone Apps) OR (App, Smartphone) OR (Apps, Smartphone) OR (Smartphone App) OR (Portable Electronic Apps) OR (App, Portable Electronic) OR (Electronic App, Portable) OR (Portable Electronic App) OR (Portable Electronic Applications) OR (Application, Portable Electronic) OR (Electronic Application, Portable) OR (Portable Electronic Application)) OR (App-based) AND TS=(randomized controlled trial or RCT or quasi-experiment or trial or intervention or “randomized clinical trial” or “controlled clinical trial”)

# Embase

#1 'stroke patient'/exp OR 'stroke patient'

#2 'cerebrovascular accident'/exp OR 'cerebrovascular accident'

#3 (strokes:ab,ti OR 'cerebrovascular accident':ab,ti OR 'cerebrovascular accidents':ab,ti OR (cva:ab,ti AND 'cerebrovascular accident':ab,ti) OR (cvas:ab,ti AND 'cerebrovascular accident':ab,ti) OR 'cerebrovascular apoplexy':ab,ti OR 'apoplexy, cerebrovascular':ab,ti OR 'vascular accident, brain':ab,ti OR 'brain vascular accident':ab,ti OR 'brain vascular accidents':ab,ti OR 'vascular accidents, brain':ab,ti OR 'cerebrovascular stroke':ab,ti OR 'cerebrovascular strokes':ab,ti OR stroke:ab,ti) AND ', cerebrovascular':ab,ti OR 'strokes, cerebrovascular':ab,ti OR apoplexy:ab,ti OR 'cerebral stroke':ab,ti OR 'cerebral strokes':ab,ti OR 'stroke, cerebral':ab,ti OR 'strokes, cerebral':ab,ti OR 'stroke, acute':ab,ti OR 'acute stroke':ab,ti OR 'acute strokes':ab,ti OR 'strokes, acute':ab,ti OR 'cerebrovascular accident, acute':ab,ti OR 'acute cerebrovascular accident':ab,ti OR 'acute cerebrovascular accidents':ab,ti OR 'cerebrovascular accidents, acute':ab,ti

#4 #1 OR #2 OR #3

#5 'mobile application'/exp

#6 'mobile applications':ab,ti OR 'application, mobile':ab,ti OR 'applications, mobile':ab,ti OR 'mobile application':ab,ti OR 'mobile apps':ab,ti OR 'app, mobile':ab,ti OR 'apps, mobile':ab,ti OR 'mobile app':ab,ti OR 'portable software apps':ab,ti OR 'app, portable software':ab,ti OR 'portable software app':ab,ti OR 'software app, portable':ab,ti OR 'portable software applications':ab,ti OR 'application, portable software':ab,ti OR 'portable software application':ab,ti OR 'software application, portable':ab,ti OR 'smartphone apps':ab,ti OR 'app, smartphone':ab,ti OR 'apps, smartphone':ab,ti OR 'smartphone app':ab,ti OR 'portable electronic apps':ab,ti OR 'app, portable electronic':ab,ti OR 'electronic app, portable':ab,ti OR 'portable electronic app':ab,ti OR 'portable electronic applications':ab,ti OR 'application, portable electronic':ab,ti OR 'electronic application, portable':ab,ti OR 'portable OR 'App-based':ab,ti

#7 #5 OR #6

#8 'randomized controlled trial':ab,ti OR rct:ab,ti OR 'quasi experiment':ab,ti OR trial:ab,ti OR intervention:ab,ti OR 'randomized clinical trial' :ab,ti OR 'controlled clinical trial ':ab,ti

#9 #4 AND #7 AND #8

# Cochrane library

#1 MeSH descriptor: [stroke] explode all trees

#2 stroke* OR “cerebrovascular accident*” OR CVA OR CVAs OR

apoplexy OR “brain vascular accident*” OR “brain infarction” OR

“cerebral infarction” OR “transient ischemic attack*” OR TIAs OR TIA

OR “cerebral hemorrhage*” OR “cerebrum hemorrhage*” OR “cerebral

parenchymal hemorrhage*” OR “cerebral brain hemorrhage*” OR

“cerebral bleeding” or “intracerebral hemorrhage*” OR ICH OR ICHs

OR “intracerebral bleeding” OR “subarachnoid hemorrhage*” OR SAH

OR SAHs OR “subarachnoid bleeding”

#3 #1 OR #2

#4 MeSH descriptor: [mobile applycation] explode all trees

#5 Mobile Applications OR “Application, Mobile” OR “Applications, Mobile” OR “Mobile Application” OR “Mobile Apps” OR “App, Mobile” OR “Apps, Mobile” OR “Mobile App” OR “Portable Software Apps” OR “App, Portable Software” OR “Portable Software App” OR “Software App, Portable” OR “Portable Software Applications” OR “Application, Portable Software” OR “Portable Software Application” OR “Software Application, Portable” OR “Smartphone Apps” OR “App, Smartphone” OR “Apps, Smartphone” OR “Smartphone App” OR “Portable Electronic Apps” OR “App, Portable Electronic” OR “Electronic App, Portable” OR “Portable Electronic App” OR “Portable Electronic Applications” OR “Application, Portable Electronic” OR “Electronic Application, Portable” OR “Portable Electronic Application” OR “App-based”

#6 #4 OR #5

#7 #3 AND #6

in Trials
